# Adherence to a treat-to-target strategy in early rheumatoid arthritis: results of the DREAM remission induction cohort

**DOI:** 10.1186/ar4099

**Published:** 2012-11-23

**Authors:** Marloes Vermeer, Hillechiena H Kuper, Hein J Bernelot Moens, Monique Hoekstra, Marcel D Posthumus, Piet LCM van Riel, Mart AFJ van de Laar

**Affiliations:** 1 Arthritis Center Twente, Department of Psychology, Health and Technology, University of Twente, PO Box 217, 7500 AE Enschede, The Netherlands; 2 Department of Rheumatology and Clinical Immunology, Medisch Spectrum Twente, PO Box 50000, 7500 KA Enschede, The Netherlands; 3 Department of Rheumatology, Ziekenhuisgroep Twente, PO Box 546, 7550 AM Hengelo, The Netherlands; 4 Department of Rheumatology, Isala Klinieken, PO Box 10500, 8000 GM Zwolle, The Netherlands; 5 Department of Rheumatology and Clinical Immunology, University Medical Center Groningen, PO Box 30001, 9700 RB Groningen, The Netherlands; 6 Department of Rheumatology, Radboud University Nijmegen Medical Centre, PO Box 9101, 6500 HB Nijmegen, The Netherlands

## Abstract

**Introduction:**

Clinical trials have demonstrated that treatment-to-target (T2T) is effective in achieving remission in early rheumatoid arthritis (RA). However, the concept of T2T has not been fully implemented yet and the question is whether a T2T strategy is feasible in daily clinical practice. The objective of the study was to evaluate the adherence to a T2T strategy aiming at remission (Disease Activity Score in 28 joints (DAS28) < 2.6) in early RA in daily practice. The recommendations regarding T2T included regular assessment of the DAS28 and advice regarding DAS28-driven treatment adjustments.

**Methods:**

A medical chart review was performed among a random sample of 100 RA patients of the DREAM remission induction cohort. At all scheduled visits, it was determined whether the clinical decisions were compliant to the T2T recommendations.

**Results:**

The 100 patients contributed to a total of 1,115 visits. The DAS28 was available in 97.9% (1,092/1,115) of the visits, of which the DAS28 was assessed at a frequency of at least every three months in 88.3% (964/1,092). Adherence to the treatment advice was observed in 69.3% (757/1,092) of the visits. In case of non-adherence when remission was present (19.5%, 108/553), most frequently medication was tapered off or discontinued when it should have been continued (7.2%, 40/553) or treatment was continued when it should have been tapered off or discontinued (6.2%, 34/553). In case of non-adherence when remission was absent (42.1%, 227/539), most frequently medication was not intensified when an intensification step should have been taken (34.9%, 188/539). The main reason for non-adherence was discordance between disease activity status according to the rheumatologist and DAS28.

**Conclusions:**

The recommendations regarding T2T were successfully implemented and high adherence was observed. This demonstrates that a T2T strategy is feasible in RA in daily clinical practice.

## Introduction

New insights into the treatment of rheumatoid arthritis (RA) have led to better care for RA patients, thereby strongly improving outcome [1,2]. Herewith, clinical remission has become the ultimate therapeutic goal in RA [3]. Several studies have demonstrated that a treatment approach including tight control of disease activity is more effective in lowering disease activity and, ultimately, reaching remission, compared with usual care [4-8]. The major keystones of such a treat-to-target (T2T) strategy are: 1) regular assessment of the disease activity using a validated outcome measure, 2) subsequent adjustments of treatment in case of persistent disease activity, preferably following a medication protocol where therapeutic consequences are predefined [9], and 3) aiming at a predefined target.

Current recommendations and guidelines on the management of RA address the importance of treating RA to a target of remission or low disease activity [3,10]. In this context, the European League Against Rheumatism (EULAR) has formulated a set of 10 international recommendations on how to achieve optimal outcomes of RA by providing guidance for T2T [11]. Although the rheumatology community worldwide endorses the importance of T2T [12], a lack of compliance with this issue is presumed in daily clinical practice. It is assumed that disease activity is not consistently measured by validated measures and medication is often not intensified or changed when disease is active in the routine care setting [6,13-15].

The implementation of guidelines and the translation of beneficial results of clinical trials to daily clinical practice are considered to be difficult [16-19]. The question is whether a T2T strategy, including protocolized treatment adjustments will be feasible in a real-life setting, which is characterized by a more heterogeneous patient population [20], variation in prescription behavior of specialists [19,21], and restriction of time, costs and resources.

In 2006, six of the Dutch Rheumatoid Arthritis Monitoring (DREAM) consortium hospitals implemented a T2T strategy in the so-called DREAM remission induction cohort. Four of these six hospitals successfully implemented the strategy and included at least 20 patients. Since the day of diagnosis, very early RA patients were treated according to a T2T strategy aiming at remission (defined as a Disease Activity Score in 28 joints (DAS28) < 2.6 [22]), including a treatment advice regarding subsequent DAS28-driven therapeutic steps [23]. The aim of the present study was to evaluate the adherence to these T2T recommendations. We examined whether these recommendations resulted in regular assessment of the disease activity with the DAS28 and whether medication was adapted according to the treatment advice. Moreover, we explored reasons for non-adherence to the T2T recommendations.

## Materials and methods

### Patients

Recommendations regarding T2T were implemented in six hospitals in The Netherlands in January 2006, as part of the DREAM remission induction cohort. Inclusion of patients into the cohort and data collection are still on-going. The cohort consists of consecutive patients newly diagnosed with RA who met the following inclusion criteria: clinical diagnosis of RA, age ≥ 18, symptom duration (defined as time from first reported symptom to diagnosis of RA by rheumatologist) of one year or less, a DAS28 ≥ 2.6, and no previous treatment with disease-modifying antirheumatic drugs (DMARDs) and/or prednisolone. Under Dutch law, this descriptive evaluation does not need approval from an ethical review board. Nonetheless, patients were fully informed, and informed consent was obtained.

For the present study, a random sample of 100 patients of the DREAM remission induction cohort was taken using the 'random sample of cases' function in SPSS. We selected patients who had a minimal follow-up of six months to ensure that every patient had at least three follow-up evaluations.

### Treatment

After inclusion, visits were scheduled at weeks 8, 12, 20, 24, 36 and 52, and every three months thereafter. Patients could visit the rheumatologist in between the scheduled cohort visits when necessary. The T2T recommendations included systematic monitoring of the disease activity with the DAS28 in combination with a treatment advice regarding predefined treatment adjustments aiming at remission (defined as a DAS28 < 2.6). Therapy consisted of initial methotrexate monotherapy (MTX), followed by the addition of sulfasalazine (SSZ) in case of non-remission, and thereafter, sulfasalazine was exchanged for anti-tumor necrosis factor (TNF)-α agents in case of moderate or high disease activity (Table 1). If the target of DAS28 < 2.6 was reached, medication was not changed. In case of sustained remission (six months or more), medication was gradually tapered and eventually discontinued. The last introduced medication was tapered off first (that is, MTX was always tapered off as the last medication). The tapering steps were as follows (all lasting six months): MTX in mg/week, 25 to 15 to 7.5 to stop; SSZ in mg/day, 3,000 to 2,000 to 1,000 to stop; adalimumab 40 mg, every week to every two weeks to stop; etanercept 50 mg, every week to every two weeks to stop; infliximab 3 mg/kg, every four weeks to every eight weeks to stop. In case of a disease flare-up (DAS28 ≥ 2.6), the most recently effective medication (dosage) was reintroduced and treatment could be subsequently intensified.

**Table 1:** Treatment protocol

Follow-up	DAS28	Medication
**Week 0**	≥ 2.6	**Methotrexate 15 mg/wk**
**Week 8**	≥ 2.6	**Methotrexate 25 mg/wk**
**Week 12**	≥ 2.6	**Methotrexate 25 mg/wk + sulfasalazine 2,000 mg/day**
**Week 20**	≥ 2.6	**Methotrexate 25 mg/wk + sulfasalazine 3,000 mg/day**
**Week 24**	≥ 3.2†	**Methotrexate 25 mg/wk + adalimumab 40 mg every 2 weeks**
**Week 36**	≥ 2.6 and decrease of > 1.2‡	**Methotrexate 25 mg/wk + adalimumab 40 mg/week**
**Week 52**	≥ 3.2†	**Methotrexate 25 mg/wk + etanercept 50 mg/week**
**1 year + 3 months**	≥ 3.2†	**Methotrexate 25 mg/wk + infliximab 3 mg/kg every 8 weeks (after a loading dose at weeks 0, 2 and 6)**
**1 year + 6 months**	≥ 2.6 and decrease of > 1.2‡	**Methotrexate 25 mg/wk + infliximab 3 mg/kg every 4 weeks**

The goal of treatment was remission (Disease Activity Score in 28 joints (DAS28) < 2.6). Treatment was intensified when this target was not met. In case of remission, medication was not changed. † Following the guidelines of the Dutch Society of Rheumatology and Dutch reimbursement regulations, anti-tumor necrosis factor α (anti-TNFα) therapy could be prescribed to patients with at least moderate disease activity (DAS28 ≥ 3.2) and in whom treatment with at least two disease-modifying antirheumatic drugs had failed (including methotrexate at 25 mg/week). ‡ Anti-TNFα therapy could be continued only if the DAS28 had decreased by > 1.2 after three months.

Deviations from the protocol were allowed on clinical indication. In patients with a sulfa allergy, SSZ was replaced by hydroxychloroquine at 400 mg per day. Nonsteroidal anti-inflammatory drugs, prednisolone ≤ 10 mg per day and intra-articular corticosteroid injections were allowed at clinical indication. Details of the protocol were reported earlier [23]. All clinical data on patient characteristics, clinical and laboratory measures, and medication use were prospectively stored in an electronic database.

### Measures

The following variables were collected at baseline: age, sex, symptom duration, fulfilment of the American College of Rheumatology (ACR) 1987 criteria for the classification of RA [24], rheumatoid factor (RF) status, anti-cyclic citrullinated peptide antibody status, C-reactive protein (CRP), patient's global assessment of pain on a 100-mm visual analogue scale (VAS), the disability index of the Dutch version of the Health Assessment Questionnaire [25,26], and component summary scores for physical and mental health of the 36-item Short Form Health Survey [27].

At every visit, joint counts were performed by trained rheumatology nurses, erythrocyte sedimentation rate (ESR) was measured and general health was filled out on a 100-mm VAS by the patient. The nurse calculated the DAS28 score and provided this, including the values of its components, to the rheumatologist.

Remission was defined as a DAS28 < 2.6. Remission according to the provisional ACR/EULAR Boolean-based definition of remission in RA was also examined, which required a tender joint count in 28 joints (TJC28) ≤ 1, swollen joint count in 28 joints (SJC28) ≤ 1, CRP ≤ 1 mg/dl and patient global assessment (PGA, on a 0 to 10 scale) ≤ 1 [28].

### Data extraction

All scheduled clinical visits were retrieved from the database and parameters for disease activity and medication were extracted. A medical chart review was performed to determine whether the clinical decisions were compliant to the above described recommendations.

Deviations from the treatment advice were classified as: not intensifying (that is, continuing/tapering off/discontinuing) instead of intensifying treatment; intensifying instead of not intensifying (that is, continuing/tapering off/discontinuing) treatment; continuing instead of tapering off/discontinuing treatment; tapering off/discontinuing instead of continuing treatment; and other deviations. We registered whether the deviations concerned conventional DMARDs or anti-TNF therapy. Possible reasons for non-adherence were retrieved from the medical chart. Medication use outside the treatment advice (including the use of prednisolone dosages > 10 mg per day) was registered.

### Statistical analysis

The primary outcome measures were the number of cohort visits in which the DAS28 was assessed and the number of these visits in which therapy was adapted according to the treatment advice, stratified by remission state (yes/no). The various deviations were reported as numbers with corresponding percentages. Pie charts were used to graphically present the extent to which the medication protocol was followed.

To test differences in the patient baseline characteristics between hospitals, we used independent *t *tests for normally distributed variables, chi-square tests for categorical variables and Mann-Whitney U tests for non-normally distributed variables.

The level of significance was set at a *P-*value < 0.05. Statistical analyses were performed using the statistical software package SPSS 18.0 (SPSS Inc., Chicago, IL, USA).

## Results

### Study sample

The baseline characteristics of the 100 patients whose medical charts were examined are presented in Table 2. This study group reflects a normal early RA population with 61.0% of the patients being female, a mean (standard deviation, SD) age of 57.7 (15.4), 61.0% RF positivity, and a mean DAS28 (SD) of 4.9 (1.1). Patients' baseline characteristics were comparable between hospitals (data not shown), except for symptom duration which differed between two hospitals; median (interquartile range, IQR) of 10.0 (6.5 to 16.0) versus 21.0 (9.8 to 30.3) weeks, *P *= 0.012. The baseline characteristics of the patients who were not included in this study did not differ significantly or clinically relevantly from the random sample of patients who were included in this study (not shown).

**Table 2:** Description of the characteristics of the 100 rheumatoid arthritis (RA) patients

**Age, mean ± SD years**	57.7 ± 15.4
**Female sex, n (%)**	61 (61.0)
**Symptom duration, median (IQR) weeks**	12.0 (8.0 to 25.0)
**Fulfillment of ACR 1987 criteria for RA, n (%)**	81/97 (83.5)
**RF positive, n (%)**	61 (61.0)
**Anti-CCP positive, n (%)**	58/98 (59.2)
**DAS28, mean ± SD**	4.9 ± 1.1
**No. of swollen joints (28 assessed), median (IQR)**	7.0 (4.0 to 12.0)
**No. of tender joints (28 assessed), median (IQR)**	4.0 (2.0 to 10.0)
**ESR, median (IQR) mm/hour**	31.2 ± 18.5
**CRP, median (IQR) mg/litre**	11.5 (5.0 to 30.8)
**Patient's assessment of general health, mean ± SD (0 to 100 VAS)**	54.0 ± 22.5
**Patient's assessment of pain, mean ± SD (0 to 100 VAS)**	51.7 ± 23.0
**HAQ score, mean ± SD**	0.9 ± 0.7
**SF-36 PCS score, mean ± SD**	38.3 ± 10.0
**SF-36 MCS score, mean ± SD**	47.8 ± 12.6

ACR, American College of Rheumatology; anti-CCP, anti-cyclic citrullinated peptide; CRP, C-reactive protein; DAS28, Disease Activity Score in 28 joints; ESR, erythrocyte sedimentation rate; HAQ, Health Assessment Questionnaire; IQR, interquartile range; MCS, mental component summary; PCS, physical component summary; RF, rheumatoid factor; SD, standard deviation; SF-36, Short-Form 36 health survey; VAS, visual analog scale

The 100 patients contributed to a total of 1,115 visits. The mean (SD) follow-up time at the time of data collection in these patients was 28.0 (10.0) months. The mean (SD) number of cohort visits per patient was 10.9 (3.6).

### Monitoring of disease activity

The DAS28 was available in 97.9% (1,092/1,115) of the scheduled cohort visits. The main reasons for the DAS28 score being missing were that the ESR was not (yet) available or the patient's general health was not assessed (data not shown). Since the level of disease activity could not be evaluated in these visits, we could not determine whether adherence to the medication protocol was accomplished. Therefore, these 23 visits (2.1%) were excluded from further analyses.

According to the T2T strategy, disease activity should be assessed using the DAS28 at least every three months. In 88.3% (964/1,092) of the visits, this recommendation was met. In the remaining 128 cases, DAS28 remission was present at the previous visit in 71.1% (91/128), and, therefore, the attending rheumatologist scheduled the next visit in six months in 93.4% (85/91) of these visits.

### Adherence treatment advice

Adherence to the treatment advice was observed in 69.3% (757/1,092) of the visits. Non-adherence at least at one visit was experienced by 91.0% of patients (91/100).

#### Remission

**Figure 1:**
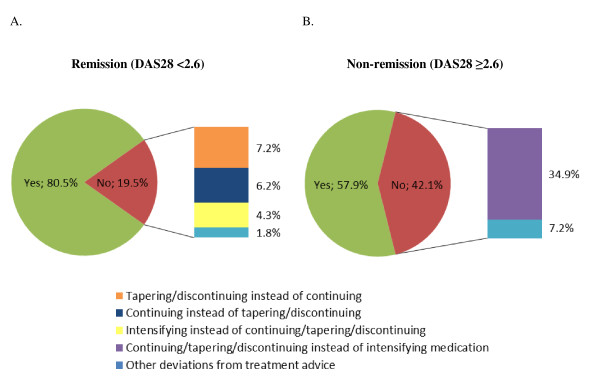
**Adherence to the treatment advice of the treat-to-target strategy**. The pie charts illustrate how often the treatment advice was followed in **A**) 553 visits in which remission (Disease Activity Score in 28 joints (DAS28) < 2.6) was present and **B**) 539 visits in which no remission (DAS28 ≥ 2.6) was present of 100 patients with early rheumatoid arthritis.

Remission was present in 50.6% (553/1,092) of the visits. The treatment advice was followed in 80.5% (445/553) of these visits, that is, medication was continued or, in case of sustained remission (DAS28 < 2.6 for at least six months), tapered off or discontinued (Figure 1A). The various deviations in the non-adherence cases (19.5%, 108/553) are also depicted in Figure 1A. Most frequently, medication was tapered off or discontinued when it should have been continued according to the treatment advice (7.2%, 40/553), or treatment was continued when it should have been tapered off or discontinued (6.2%, 34/553). Furthermore, medication was intensified in 4.3% (24/553) in spite of DAS28 remission. In 9 of these 24 cases, the patient had at least one swollen joint. Other deviations were observed in 1.8% (10/553). In 12.0% (13/108) of these non-adherence cases, the deviation concerned anti-TNF therapy.

The reasons for non-adherence are presented in Table 3. The most frequently observed reasons for non-adherence were the presence of active disease according to the rheumatologist (that is, based on the presentation of the patient's overall disease activity, in particular, the degree of arthritis) and drug side effects.

**Table 3:** Reasons for non-adherence to the treatment advice of the treat-to-target strategy

	Remission (DAS28 < 2.6) (*n *= 553)	Non-remission (DAS28 ≥ 2.6) (*n *= 539)
**Tapering off/discontinuing instead of continuing**	40 (7.2)	
Unknown	12	
Patient wish	3	
Side effects	19	
Clinical remission	5	
Other	1	
**Continuing instead of tapering off/discontinuing**	34 (6.2)	.
Unknown	23	
Patient wish	4	
Active disease	5	
Other	2	
Intensifying instead of not intensifying*	24 (4.3)	.
Unknown	2	
Patient wish	2	
Active disease	20	
Not intensifying* instead of intensifying	.	188 (34.9)
Unknown		29
Patient wish		13
Side effects		32
Clinical remission		106
Other		8
**Other deviations from treatment advice**	10 (1.8)	39 (7.2)
Unknown	5	10
Patient wish	.	2
Side effects	4	16
Clinical remission	.	2
Other	1	9

Data are presented of a total of 1097 visits of 100 patients with early rheumatoid arthritis, stratified by remission state according to the Disease Activity Score in 28 joints (DAS28). Values regarding the type of deviation are presented as number (%), values regarding the reasons for non-adherence are presented as number. *Including continuing, tapering off and discontinuing medication.

Remission according to the provisional ACR/EULAR definition of remission was observed in 42.9% (237/524) of these visits (remission could not be evaluated in all visits due to missing values for CRP).

#### Non-remission

In case of non-remission, the treatment advice was followed in 57.9% (312/539) of the visits (Figure 1B), meaning that therapy was intensified. Figure 1B also shows the various deviations in case of non-adherence (42.1%, 227/539). The most frequently observed deviation was that medication was not intensified (that is, continued, tapered off or discontinued) when an intensification step should have been taken according to the treatment advice (34.9%, 188/539). Other deviations were observed in 7.2% (39/539). In 21.6% (49/227) of the non-adherence cases, the deviation concerned anti-TNF therapy.

In the non-adherence cases, disease activity was low (2.6 ≤ DAS28 ≤ 3.2) in 43.2% (98/227), moderate (3.2 < DAS28 ≤ 5.1) in 49.8% (113/227), and high (DAS28 > 5.1) in 7.0% (16/227).

Table 3 presents the reasons for non-adherence. The most frequently observed reason for deviation was that clinical remission was present according to the rheumatologist, even though the DAS28 was 2.6 or higher. In these 108 visits, the distribution of the clinical variables was as follows: median (IQR) SJC28 of 1.0 (0.0 to 3.0), median (IQR) TJC28 of 0.0 (0.0 to 3.0), mean (SD) ESR of 22.6 (14.9), and median (IQR) CRP of 5.0 (4.0 to 8.0). The mean (SD) DAS28 was 3.3 (0.7) and 54.6% (59/108) of these patients had a DAS28 ≤ 3.2. The mean (SD) scores of the patient's assessment of pain was 31.6 (21.4) and of general health 34.6 (21.1).

Non-remission according to the provisional ACR/EULAR definition of remission was observed in 94.2% (508/514) of these visits (remission could not be evaluated in all visits due to missing values for CRP).

#### Other medication

Disease modifying medication outside the treatment advice was prescribed in 8.0% (8/100) of the patients; 1 patient received leflunomide and 7 patients received at least one intra-muscular corticosteroid injection. Prednisolone dosages > 10 mg per day were given to 7.0% (7/100) of the patients at least at one visit.

#### Drug side effects

Adherence to the treatment advice was prevented by drug side effects in 71 visits. Table 4 presents the various side effects that were registered in the medical charts. If the patient experienced more than one side effect, we listed only the first reported side effect.

**Table 4:** Drug side effects (*n *= 71)

**Not defined**	23 (32.4)
**Abnormal liver function tests**	12 (16.9)
**Nausea, abdominal pain or diarrhoea**	10 (14.1)
**Haematological abnormalities**	7 (9.9)
**Pulmonary problem**	7 (9.9)
**Mental or mood changes**	5 (7.0)
**Hair loss**	3 (4.2)
**Skin rash**	2 (2.8)
**Vision problem**	2 (2.8)

Values are presented as number (%).

## Discussion

This descriptive evaluation demonstrated that a T2T strategy is possible in daily clinical practice. The recommendations regarding T2T were successfully implemented in the participating rheumatology clinics of the DREAM remission induction cohort. Disease activity was regularly and systematically measured using the DAS28 and adherence to the treatment advice was high. In case of non-adherence to the recommendations, valid arguments for deviating were observed in the majority of these cases.

Real-life observational data regarding the adherence to a T2T strategy in daily clinical practice were analyzed. These are valuable data because T2T strategies are often conducted in the setting of randomized controlled trials (RCTs), which entails a more controlled environment compared to routine care. This was a retrospective evaluation and, therefore, the attending physicians were not influenced by the goal of this analysis.

The successful implementation of T2T in the DREAM hospitals can be explained by several factors. First, the evidence from RCTs and, subsequently, the fact that current guidelines and recommendations underline the importance of T2T has raised rheumatologists' awareness about the effectiveness of T2T. Second, the recommendations regarding the frequency of monitoring and the therapeutic regimen in the present study were fit in as close as possible with the conventional management of RA in daily clinical practice. Prior to the implementation of T2T, consensus was reached on the recommendations by all rheumatologists of the participating hospitals. Third, the organization of care was arranged as such that the treatment approach did not require extra effort and time from the rheumatologist during the clinical visit. Prior to the visit to the rheumatologist, RA disease activity according to the DAS28 was evaluated by a trained rheumatology nurse. The rheumatology nurses take part in annual DAS28 training sessions, which guarantee the uniformity of the DAS28 assessment. A previous study by Van Hulst *et al*. suggests that nurse-led care, including DAS28 measurement, may be helpful in making DAS28 assessments more feasible for daily clinical practice settings [13]. Furthermore, general practitioners are aware of the importance of early referral of patients with symptoms of arthritis to the rheumatologist, thereby making an early diagnosis possible.

At a percentage of 69%, the observed level of adherence to the T2T recommendations was probably optimal, as striving for 100% adherence is not realistic because treatment of patients is subject to side effects and comorbidities. Moreover, it is obvious that patients are involved in their treatment decision making process. Previous studies have shown that discordance exists between the patients' and rheumatologists' rating of disease activity [29,30] and that they approach the decision to intensify medication differently [31,32]. Moreover, patients may be reluctant to change medications frequently and fear side effects. In the present study, the main reason for non-adherence was discordance between disease activity status according to the rheumatologist and the DAS28, which might be explained by properties of the DAS28 algorithm. The feet are omitted in the DAS28; however, disease activity in the foot joints is frequently observed in RA, even in patients who are considered to be in DAS28 remission [33]. This among other factors has led to the debate of whether the cut-off point for DAS28 remission reflects true clinical remission [34,35]. In the therapeutic decision making, rheumatologists do not always rely solely on the DAS28 [36], but also other markers of inflammation and/or progression [31,37] and patients' characteristics [38] are taken into account. The DAS28 is suggested to be a tool to guide decision making in RA; nevertheless, it cannot always replace the clinical judgement in the context of an individual patient. Therefore, T2T should be performed with thoughtful consideration.

This study has some drawbacks. First, adherence to the recommendations of the T2T strategy was evaluated in only 100 patients of the total cohort, which includes more than 700 patients at the time of data collection. However, a random sample was taken which we believe was representative for the total cohort. Second, it was not explicitly requested to report the reason for protocol deviations, and, therefore, not all reasons could be retrieved. Third, this T2T strategy reflects the effects of only one medication strategy; no other treatment strategies were included. Several therapeutic regimes and treatment approaches have been introduced over the last decade, but the most optimal strategy for patients newly diagnosed with RA remains undecided. Moreover, after the initiation of the DREAM remission induction cohort, it emerged that the dose increase of anti-TNF therapy might have limited effectiveness [39] and also the effectiveness of a third anti-TNF agent in case of failure of two previous anti-TNF agents has been debated. This might have led to deviations from the advised anti-TNF therapy steps. Fourth, it was not investigated whether failure to be adherent to the strategy had any impact on whether patients achieved remission.

## Conclusions

In conclusion, this study showed that the implementation of T2T is feasible in very early RA in daily clinical practice. We demonstrated a high adherence to the T2T recommendations, which comprised regular assessment of the DAS28 and treatment advice regarding subsequent DAS28-driven therapeutic steps.

## Abbreviations

ACR: American College of Rheumatology; Anti-CCP: anti-cyclic citrullinated peptide; Anti-TNF: anti-tumor necrosis factor; CRP: C-reactive protein; DAS28: Disease Activity Score in 28 joints; DMARDs: disease-modifying antirheumatic drugs; DREAM: Dutch Rheumatoid Arthritis Monitoring; ESR: erythrocyte sedimentation rate; EULAR: European League Against Rheumatism; HAQ: Health Assessment Questionnaire; IQR: interquartile range; MCS: mental component summary; MTX: methotrexate; PCS: physical component summary; PGA: patient global assessment; RA: rheumatoid arthritis; RCTs: randomized controlled trials; RF: rheumatoid factor; SD: standard deviation; SF-36: Short-Form 36 health survey; SJC28: swollen joint count in 28 joints; SSZ: sulfasalazine; T2T: treat-to-target; TJC28: tender joint count in 28 joints; TNF: tumor necrosis factor; VAS: visual analogue scale.

## Competing interests

The authors declare that they have no competing interests.

## Authors' contributions

MV contributed to the design of the study, data collection, analysis and interpretation, and drafted the manuscript. HHK contributed to the design of the study, data collection and interpretation, and drafted the manuscript. HJBM, MH and MDH contributed to data collection and interpretation, and critically revised the manuscript. PLCMvR contributed to the design of the study, data interpretation, and critically revised the manuscript. MvdL contributed to the design of the study, data collection and interpretation, and drafted the manuscript. All authors read and approved the final manuscript.
